# Combined treatment modality for intracranial germinomas: results of a multicentre SFOP experience

**DOI:** 10.1038/sj.bjc.6690192

**Published:** 1999-03

**Authors:** E Bouffet, M C Baranzelli, C Patte, M Portas, C Edan, P Chastagner, F Mechinaud-Lacroix, C Kalifa, on behalf of the Société Française d'Oncologie Pédiatrique

**Affiliations:** Service d'Oncologie Pédiatrique, Centre L Bérard, 28 rue Laennec, Lyon Cédex, 69008, France; Service d'Oncologie Médicale <A>, Centre O Lambret, 3 rue F Combemale, Lille Cédex, 59020, France; Service de Pédiatrie, Institut Gustave Roussy, 39 rue C Desmoulins, Villejuif Cédex, 94805, France; Service d'Oncologie Pédiatrique, Hôpital d'Enfants de la Timone, Marseille Cédex, 13385, France; Clinique Médicale Infantile, CHR, 16 rue de Bulgarie, Rennes Cédex, 35056, France; Service de Médecine Infantile II, Hôpital d'Enfants, Vandoeuvre, France; Service d'Oncologie Pédiatrique, CHR, Nantes Cédex, 44035, France

**Keywords:** germinoma, tumour markers, chemotherapy, local irradiation, bifocal tumour

## Abstract

Conventional therapy for intracranial germinomas is craniospinal irradiation. In 1990, the Société Française d'Oncologie Pédiatrique initiated a study combining chemotherapy (alternating courses of etoposide–carboplatin and etoposide–ifosfamide for a recommended total of four courses) with 40 Gy local irradiation for patients with localized germinomas. Metastatic patients were allocated to receive low-dose craniospinal radiotherapy. Fifty-seven patients were enrolled between 1990 and 1996. Forty-seven had biopsy-proven germinoma. Biopsy was not performed in ten patients (four had diagnostic tumour markers and in six the neurosurgeon felt biopsy was contraindicated). Fifty-one patients had localized disease, and six leptomeningeal dissemination. Seven patients had bifocal tumour. All but one patient received at least four courses of chemotherapy. Toxicity was mainly haematological. Patients with diabetus insipidus (*n* = 25) commonly developed electrolyte disturbances during chemotherapy. No patient developed tumour progression during chemotherapy. Fifty patients received local radiotherapy with a median dose of 40 Gy to the initial tumour volume. Six metastatic patients, and one patient with localized disease who stopped chemotherapy due to severe toxicity, received craniospinal radiotherapy. The median follow-up for the group was 42 months. Four patients relapsed 9, 10, 38 and 57 months after diagnosis. Three achieved second complete remission following salvage treatment with chemotherapy alone or chemo-radiotherapy. The estimated 3-year survival probability is 98% (CI: 86.6–99.7%) and the estimated 3-year event-free survival is 96.4% (CI: 86.2–99.1%). This study shows that excellent survival rates can be achieved by combining chemotherapy and local radiotherapy in patients with non-metastatic intracranial germinomas. © 1999 Cancer Research Campaign


*British Journal of Cancer* (1999) **79**(7/8), 1199–1204
© 1999 Cancer Research Campaign
Article no. bjoc.1998.0192

### Combined treatment modality for intracranial germinomas: results of a multicentre SFOP experience

**E Bouffet[1,*], MC Baranzelli[2], C Patte[3], M Portas[4], C Edan[5], P Chastagner[6], F Mechinaud-Lacroix[7], C Kalifa[3] on behalf of the Société Française d'Oncologie Pédiatrique**

[1]Service d'Oncologie Pédiatrique, Centre L Bérard, 28 rue Laennec, 69008 Lyon Cédex, France; [2]Service d'Oncologie Médicale ‹ A ›, Centre O Lambret, 3 rue F Combemale, 59020 Lille Cédex, France; [3]Service de Pédiatrie, Institut Gustave Roussy, 39 rue C Desmoulins, 94805 Villejuif Cédex, France; [4]Service d'Oncologie Pédiatrique, Hôpital d'Enfants de la Timone, 13385 Marseille Cédex, France; [5]Clinique Médicale Infantile, CHR, 16 rue de Bulgarie, 35056 Rennes Cédex, France; [6]Service de Médecine Infantile II, Hôpital d'Enfants, Vandoeuvre, France; [7]Service d'Oncologie Pédiatrique, CHR, 44035 Nantes Cédex, France

**Summary** Conventional therapy for intracranial germinomas is craniospinal irradiation. In 1990, the Société Française d'Oncologie Pédiatrique initiated a study combining chemotherapy (alternating courses of etoposide–carboplatin and etoposide–ifosfamide for a recommended total of four courses) with 40 Gy local irradiation for patients with localized germinomas. Metastatic patients were allocated to receive low-dose craniospinal radiotherapy. Fifty-seven patients were enrolled between 1990 and 1996. Forty-seven had biopsy-proven germinoma. Biopsy was not performed in ten patients (four had diagnostic tumour markers and in six the neurosurgeon felt biopsy was contraindicated). Fifty-one patients had localized disease, and six leptomeningeal dissemination. Seven patients had bifocal tumour. All but one patient received at least four courses of chemotherapy. Toxicity was mainly haematological. Patients with diabetus insipidus ($n$ = 25) commonly developed electrolyte disturbances during chemotherapy. No patient developed tumour progression during chemotherapy. Fifty patients received local radiotherapy with a median dose of 40 Gy to the initial tumour volume. Six metastatic patients, and one patient with localized disease who stopped chemotherapy due to severe toxicity, received craniospinal radiotherapy. The median follow-up for the group was 42 months. Four patients relapsed 9, 10, 38 and 57 months after diagnosis. Three achieved second complete remission following salvage treatment with chemotherapy alone or chemo-radiotherapy. The estimated 3-year survival probability is 98% (CI: 86.6–99.7%) and the estimated 3-year event-free survival is 96.4% (CI: 86.2–99.1%). This study shows that excellent survival rates can be achieved by combining chemotherapy and local radiotherapy in patients with non-metastatic intracranial germinomas.

**Keywords**: germinoma; tumour markers; chemotherapy; local irradiation; bifocal tumour

Primary central nervous system germ cell tumours (CNS GCTs) are relatively rare and make up less than 3% of all brain tumours. Because of the rarity of CNS GCTs, published series usually combine both germinoma and non-germinomatous intracranial germ cell tumours. Germinoma is a specific entity, however, and deserves special consideration in terms of management. At least half to two-thirds of CNS GCTs are germinomas. Like its counterpart in the testis, this tumour is one of the most radiosensitive. Intracranial germinoma can be cured with radiotherapy; event-free and overall survival using standard craniospinal radiotherapy ranges between 70 and 95% (Sung et al, 1978; Linstadt et al, 1988; Shibamoto et al, 1988; Dearnaley et al, 1990). Despite this high cure rate, the treatment of germinoma has generated much interest and given rise to extensive controversies in recent years. Despite its effectiveness, craniospinal radiotherapy is responsible for late effects such as cognitive dysfunction, vascular pathology, endocrinopathy, spinal growth impairment and leukoencephalopathy (Darendeliler et al, 1990; Clayton et al, 1991; Mulhern et al, 1992).

It has been shown that, like seminoma of the testis or dysgerminoma of the ovary, intracranial germinomas are also chemosensitive. Using systemic chemotherapy, promising response rates have been achieved in newly diagnosed and relapsing patients with cyclophosphamide (Allen et al, 1987) or cisplatin-based regimens (Kobayashi, 1989; Patel et al, 1992). Consequently, new treatment strategies have been developed to use primary chemotherapy to avoid or to reduce radiotherapy in good responders. In 1990, The Société Française d'Oncologie Pédiatrique (SFOP) initiated a multicentre study to replace prophylactic craniospinal radiotherapy with systemic chemotherapy and to restrict radiotherapy to a focal boost of 40 Gy to the initial tumour volume in children with non-metastatic germinomas. Children with metastatic germinomas were allocated to receive chemotherapy and low-dose craniospinal radiotherapy. This paper reports the results of this strategy in 57 patients with intracranial germinomas treated between January 1990 and December 1996.

### PATIENTS AND METHODS

The pathological diagnosis of germinoma was required before initiation of the protocol. Patients with alpha-fetoprotein (αFP) and/or human chorionic gonadotrophin (HCG) and/or beta human chorionic gonadotrophin (βHCG) secretion were treated according to the SFOP protocol for intracranial secreting germ tumours. However, patients with moderate secretion of βHCG (50 ml U $ml^{-1}$) detected in serum or cerebrospinal fluid (CSF) were eligible since germinomatous tumours may present with a moderate secretion

*Received 2 March 1998*
*Revised 13 July 1998*
*Accepted 3 August 1998*

*Correspondence to:* MC Baranzelli, Service d'Oncologie Médicale ‹A›, Centre O Lambret, 3 rue F Combemale, 59020 Lille Cédex, France

*Present address: Department of Paediatric Oncology, Royal Marsden Hospital, Sutton, Surrey SM2 5PT, UK

when they contain scattered syncytiotrophoblastic giant cells (Mostofi et al, 1988; Munperow et al, 1992). For these patients with moderate secretion, histological confirmation of germinoma was recommended but not compulsory. Six patients presenting with pineal (two patients), suprasellar (three patients) or bifocal (one patient) tumour with negative markers and without histological diagnosis are detailed separately. Initial staging procedure included clinical examination, assessment of tumour markers in the serum and/or the CSF, review of the operative notes, postoperative cranial computed tomography (CT) or magnetic resonance imaging (MRI) scan, myelogram or MRI scan of the spine and cytological CSF examination.

After diagnosis, patients were given four courses of chemotherapy. The regimen was administered as follows: Courses 1 and 3: carboplatin (600 mg $m^{-2}$) on day 1; etoposide (150 mg $m^{-2}$ $day^{-1}$) from days 1 to 3; Courses 2 and 4: ifosfamide (1.8 g $m^{-2}$ $day^{-1}$) from days 21 to 25 and etoposide (150 mg $m^{-2}$ $day^{-1}$) from days 21 to 23. Response was assessed after two courses by repeating those diagnostic tests that originally identified the evaluable disease. After the four courses of chemotherapy, radiation therapy was administered to the initial tumour volume, at a daily dose of 1.8 Gy with 5 weekly fractions up to a recommended dose of 40 Gy delivered over 4.5 weeks. Radiotherapy guidelines recommended using a linear accelerator of 4.6 or 9 MeV with at least two parallel opposed fields. The recommended safety margins were 2 cm around the initial tumour. In case of disseminated germinoma, craniospinal radiotherapy was added to the protocol. The recommended dose to the brain and the spine was 25–30 Gy with a 10 Gy boost on metastatic deposits. However, local rather than craniospinal radiotherapy was recommended for patients with non-metastatic bifocal tumours.

All patients were followed clinically during and after treatment. CT or MRI scan of the head with and without administration of contrast material, was obtained 2 months after the completion of the radiotherapy, and then every 4 months during the first 2 years unless additional examinations were indicated clinically. The scans were then repeated at 6-monthly intervals. Because originally non-secreting patients may relapse with αFP and/or βHCG secretion (Ono et al, 1994), tumour markers were regularly checked. Progression was defined as any new lesion detected in any of the examinations with or without elevation of tumour markers. A complete restaging (CSF examination, CT scan or MRI of the brain, and MRI scan of the spine, tumour markers) was performed for relapsing patients.

### RESULTS (Table 1)

Between January 1990 and December 1996, 99 newly diagnosed patients from 25 centres were enrolled in the SFOP protocol for intracranial GCTs. Fifty-nine were registered in the germinoma study. Other patients were registered in the non-germinomatous intracranial GCT study. Patients with a biopsy-proven germinoma associated with high βHCG secretion and/or αFP secretion were allocated to the latter study group. Out of the group of 59 patients with germinomas, two non-metastatic patients received chemotherapy followed by craniospinal radiotherapy due to physician decision and are not included in this report. Fifty-seven patients treated according to the SFOP protocol are reported here. Forty-seven of them were included on the basis of histological diagnosis. Ten were treated without having a biopsy either because of a moderate elevation of βHCG (less than 50 ml U/ml, four patients) or because the neurosurgeon was reluctant to perform a biopsy (six patients). Six patients had metastatic disease at presentation: two presented with positive CSF cytology (assessed by lumbar puncture), two had spinal metastases and two had diffuse periventricular seeding.

**Table 1** Patients' characteristics

| | |
|---|---|
| Number of patients | 57 |
| Male | 43 |
| Female | 14 |
| Age at diagnosis | |
| Median | 13.5 |
| Range | 4.1–22 |
| Tumour location | |
| Pineal | 20 |
| Suprasellar | 28 |
| Thalamic | 2 |
| Bifocal | 7 |
| Leptomeningeal disease at diagnosis | 6 |
| Diagnostic procedure | |
| Resection | 18 |
| Biopsy | 29 |
| Tumour marker only | 4 |
| None | 6 |
| βHCG secretion | 9 |

The median age was 13.5 years (range 50 months to 22 years). There were 43 males and 14 females. Fifty patients had unifocal lesions, the site of origin of the tumour being suprasellar in 20, pineal in 28, thalamic in two. Seven patients had bifocal tumours, which in all combined a suprasellar and a pineal mass. These patients – apart from one with concomitant spinal deposits – were not considered as metastatic and received the treatment for non-metastatic patients. Twenty-nine patients with biopsy-proven localized germinoma have been the subject of a preliminary analysis (Baranzelli et al, 1997).

### Surgery

Forty-seven patients had surgery. This was limited to a stereotactic biopsy in 22 patients or an open biopsy in seven. Eighteen patients had a more aggressive surgery with either partial (12 patients) or total (six patients) resection.

### Chemotherapy administration and response

Fifty-two patients received four courses of chemotherapy according to the protocol. One patient developed severe toxicity after the first course and chemotherapy was therefore suspended. He consequently received conventional craniospinal radiotherapy instead of local radiotherapy. Four patients had protocol deviations and received five, eight, nine and 12 courses of chemotherapy, respectively. Response to chemotherapy was reported after two and four courses in 38 out of 51 evaluable patients who started chemotherapy with an assessable mass. All patients responded. Following four courses, complete response was observed in 18 patients, and 20 patients demonstrated residual abnormalities, mainly mild focal enhancement or calcified debris. None of these patients had second surgery on the residue.

Radiotherapy was given according to the protocol in 55 patients. The median time from diagnosis to the starting day of radiotherapy was 107 days (range 88–159 days) for those patients who received four courses of chemotherapy. The six patients with metastatic

 © Cancer Research Campaign 1999

germinoma received craniospinal radiotherapy, and one patient received craniospinal radiotherapy due to severe toxicity after one course of chemotherapy. Forty-eight patients received local treatment using conventional radiotherapy at a median dose of 40 Gy. Eight patients received a dose greater than 40 Gy to the primary. One patient with congenital panhypopituitarism received stereotactic radiotherapy.

### Toxicity

The median interval between courses ranged from 23 days (18–51 days) for cycle 1 to 24 days (19–41 days) for cycle 3. Data on haematopoietic toxicity are available for 230 courses. Grade 3 or 4 WHO toxicity (platelet count $< 50 \times 10^9\ l^{-1}$ and/or neutrophil count $< 0.5 \times 10^9\ l^{-1}$) occurred in 106 courses, and was similar following either the carboplatin-containing courses (56 episodes) or the ifosfamide containing ones (50 episodes). Six patients had bacterial septicaemia, one episode occurring after course 1, leading to cessation of chemotherapy.

The major non-haematopoietic toxicity was electrolyte imbalance during courses 1, 2 and 4 in patients with diabetes insipidus. All patients with diabetes insipidus ($n$ = 25) experienced electrolyte disturbances, which required prolonged hospitalization in five patients following course 1, five patients following course 2 and two patients following course 4. Four patients presented with grade 3 neurological toxicity during ifosfamide administration. Grade 2–3 gastrointestinal toxicity, including abdominal pain and diarrhoea, was reported in 11 courses. No toxic death occurred during chemotherapy or radiotherapy.

### Outcome (Figure 1)

The median follow-up time for the 57 patients was 42 months (range 6–90 months). The estimated 3-year survival probability is 98% [confidence interval (CI): 86.6–99.7%] and the estimated 3-year event-free survival is 96.4% (CI: 86.2–99.1%). Overall and event-free survival are shown in Figure 1. No patient experienced treatment failure while on therapy. Four patients developed tumour recurrence 9, 10, 38 and 57 months after diagnosis. Two patients had raised βHCG at the time of recurrence, including one who had no tumour marker at the time of diagnosis. All recurrences had at least an intracranial component, involving the trigone and the cerebellum in one patient, the third ventricle in the second, the pineal area in the third, and the pineal area and the lateral ventricle in the last patient. One initially metastatic patient had a spinal deposit on the MRI scan. Cytological examination of the CSF was positive in another patient. Salvage therapy was given at the time of recurrence in all patients. One achieved transient response to a cisplatin–ifosfamide–etoposide combination but ultimately progressed and died 6 months post-recurrence. One patient was retreated with an etoposide–carboplatin combination followed by craniospinal radiotherapy. He is currently in second complete remission, 22 months post-recurrence. Two patients achieved second remission with a cisplatin–ifosfamide–etoposide combination. They subsequently received high-dose chemotherapy followed by peripheral stem cell transplants, and are in second complete response 8 and 24 months post-recurrence, respectively. Overall, three patients with initial localized disease relapsed and achieved second remission. One metastatic patient relapsed and died of disease. No recurrence was observed among non-metastatic patients with bifocal tumours.

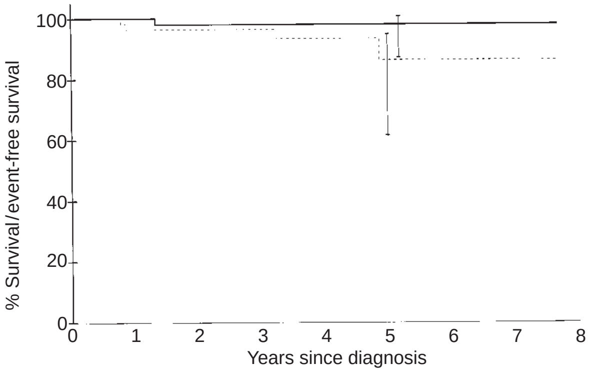

**Figure 1** Event-free (– – –) and overall (——) survival

### Outcome of patients who did not have surgery at the time of diagnosis

Ten patients did not undergo initial surgery. Nine received four courses of chemotherapy, and one received five courses. The four patients with raised βHCG all achieved biological remission after one course of chemotherapy. CT or MRI scan assessment after four courses showed complete response in seven patients and partial response in three. Radiotherapy was given as per protocol in all patients. Two patients relapsed. One had a loco-regional relapse (pineal area and trigone) 10 months post-diagnosis. He underwent a biopsy, which confirmed the diagnosis of germinoma. He achieved complete remission following chemotherapy and craniospinal radiotherapy and is currently free of disease 22 months post recurrence. The second patient initially had low-level secretion of βHCG. He relapsed in the cerebellum and the trigone 57 months post-diagnosis. He achieved complete remission after four courses of a cisplatin–ifosfamide–etoposide regimen and then received high-dose chemotherapy with stem cell rescue. He is currently in complete remission 8 months after recurrence.

### DISCUSSION

Despite achievement of high cure rates, the current management of intracranial germinomas remains controversial (Oi et al, 1992; Matsutani et al, 1997). Several aspects of the management differ between institutions or national groups. Surgery as an initial procedure is strongly supported by the majority of neurosurgeons in America and Europe, but numerous Asian and African institutions still favour the ‘radiotherapeutic test’ (Oi et al, 1992). Craniospinal radiotherapy is still the standard treatment for many centres, but some radiotherapists favour local radiotherapy and oncologists are increasingly interested in giving chemotherapy in order to reduce either the dose or the extent of radiotherapy. The SFOP group has prospectively explored the feasibility of a combined approach that can be compared to other prospective multicentre trials such as the MAKEI trials (Gobel et al, 1989, 1993) and the First International Central Nervous System Germ Cell Tumor Study (Balmaceda et al, 1996).

In germinoma, there is little evidence that the extent of surgery influences the outcome. Reports that favour radical surgery combine in their analysis germinomas and non-germinomatous GCTs (Matsutani et al, 1997). In our series, all four relapses occurred in patients who had either incomplete resection or no surgery. However, only six patients had complete removal of their

© Cancer Research Campaign 1999

tumour and the low incidence of recurrences (four out of 51 who had residual disease at initiation of chemotherapy) does not support a role for aggressive debulking. Sawamura et al (1997) advocated limited biopsy in germinomas, which provides sufficient information for diagnosis and treatment planning. Some patients may present with a slight isolated elevation of HCG or βHCG in serum and/or CSF of below 50 ml U ml. Such levels are highly suggestive of germinomas with syncytiotrophoblastic cells (Uematsu et al, 1992; Shibamoto et al, 1997). For these patients a diagnostic biopsy is questionable given the theoretical risk of morbidity resulting from surgical procedures. In our series, four out of nine patients who presented with a slight isolated elevation of HCG had no histological diagnosis and their tumour evolved in the same manner with the same treatment. Moreover, six patients were treated on imaging criteria without either histological proof or any elevation of βHCG favouring the diagnosis of germinoma with syncytiotrophoblastic cells. In each of these cases, biopsy was not performed because of concerns of the neurosurgeon. The decision to include patients without histological diagnosis in our final analysis may be controversial. However, all these patients did respond to chemotherapy and all but two achieved long-term remission with the treatment plan. One patient underwent a biopsy at the time of relapse, which ultimately confirmed the diagnosis of germinoma. A ‘chemotherapeutic test’ for patients with a high risk of surgical morbidity is, in our experience, a successful alternative to the ‘radiotherapeutic test’. One may hope that detection of sensitive and specific tumour markers for germinomas will contribute to non-invasive diagnosis (Shinoda et al, 1988). Some authors have suggested that intracranial germinoma with syncytiotrophoblastic giant cells producing HCG is associated with a higher recurrence rate compared with pure germinoma (Uematsu et al, 1992; Shibamoto et al, 1997). Only one patient relapsed in the group of nine who had slight elevation of HCG. This might suggest that highly effective treatment can mask this adverse prognostic feature, or that this group needs a longer follow-up to draw definite conclusions, since late recurrences have been reported with this entity.

Using craniospinal radiotherapy, the survival rate in large series ranges between 75 and 95%. These good results have provided the rationale for a systematic approach using craniospinal radiotherapy in all patients, regardless of their metastatic status. However, this treatment was developed at a time when staging procedures were limited, and it was therefore a safe option for unstaged patients. With modern imaging procedures, the proportion of patients presenting with metastatic disease at the time of diagnosis is low, and the risk of secondary spinal seeding in germinoma does not exceed 15% in large series (Sung et al, 1978; Linstadt et al, 1988; Matsumani et al, 1997). Furthermore, in a review of the literature, Brada et al (1990) have shown that the incidence of secondary seeding did not differ between patients treated with craniospinal radiotherapy and whole brain radiotherapy. The benefit of craniospinal radiotherapy thus seems questionable (Linstadt et al, 1988). Recent studies have suggested that reduced doses of prophylactic craniospinal radiotherapy are effective (Hardenbergh et al, 1997; Plowman et al, 1997). Single institution experiences have shown that, with complete diagnostic craniospinal evaluation, spinal irradiation is not necessary and cure rates for localized germinomas are excellent with focal irradiation (Wolden et al, 1995; Sawamura et al, 1998). When focal radiotherapy is advocated, the dimension of the field is another matter of debate. Some authors recommend irradiation to a generous local field encompassing the tumour site for localized germinomas (Dattoli et al, 1990; Shibamato et al, 1994). For other authors, the local field should include the third and lateral ventricles as well as the sellar and the pineal region (Matsuani et al, 1997; Shirato et al, 1997). New techniques such as radiosurgery or stereotactic radiotherapy have been proposed (Manera et al, 1996). The optimal dose of radiotherapy to the tumour bed is still unclear. Most authors recommend 40 Gy, but some authors consider that 50–60 Gy is the adequate dose for this tumour, leading to better local control (Wolden et al, 1995; Haddock et al, 1997). In our experience, all recurrences had an intracranial component, and this could argue for a larger field or higher doses of radiotherapy. The small number of failures with our present approach and the risk of increased morbidity with larger fields or higher doses of radiotherapy in this young population point out the difficulty of achieving a consensus. Conversely, the dose of 40 Gy proposed in the present protocol could perhaps be reduced. This choice was deliberate following the disappointing results observed in a previous SFOP study using a 30 Gy boost to the tumour bed (Calaminus et al, 1994). The chemotherapy regimen in this latter protocol was, however, significantly different and definitely less effective. Our series shows that chemotherapy followed by local radiotherapy delivered to the initial tumour volume is a safe alternative to craniospinal radiotherapy or whole ventricle irradiation.

Long-term effects of craniospinal radiotherapy are well known, and mainly affect young children. Those who favour craniospinal radiotherapy argue that patients with germinoma are older than patients with medulloblastoma, and neuropsychologic disturbances or spinal growth alterations seem to be less marked in this age range (Kiltie et al, 1995). However, eighteen out of 57 children in our series were less than 12 years old but 11 additional children above 12 years of age presented with either isolated growth hormone deficiency or panhypopituitarism and, therefore, short stature at the time of diagnosis. The risk of short stature following craniospinal radiotherapy has consequently to be regarded as an important issue regardless of the age at presentation.

We favoured a combination of chemotherapy and local radiotherapy for patients with localized germinomas. Platinum compounds are effective in intracranial GCTs, with high response rates reported in germinoma and non-germinomatous tumours using cisplatin or carboplatin (Kobayashi, 1989; Patel et al, 1992; Allen et al, 1994). The carboplatin–etoposide association has demonstrated impressive response rates in embryonal tumours of the CNS (Gentet et al, 1994; Heideman et al, 1995). Ifosfamide is a cyclophosphamide analogue, which is commonly used for poor risk extracranial GCTs (Roth, 1996). Cyclophosphamide has shown promising activity in intracranial GCTs, particularly in germinomas with 100% responses among 11 newly diagnosed patients in the pilot study reported by Allen et al (1985, 1987). Data on ifosfamide efficacy in intracranial GCTs are few, but this drug has shown excellent CSF diffusion (Ninane et al, 1989). In our experience, all assessable patients achieved at least partial response after two courses of chemotherapy, but no specific conclusion can be made from the etoposide–ifosfamide combination. It is noteworthy that bleomycin was not used in this study. The toxicity of this agent, particularly in children, and the lack of data on its efficacy in intracranial GCTs have precluded its use in our study. The response rate to chemotherapy is excellent since all patients with evaluable disease achieved either partial or total response after chemotherapy. The toxicity of this regimen is limited, and no patient died from a chemotherapy-related complication. Apart from haematological

 © Cancer Research Campaign 1999

toxicity, the main complication was destabilization of diabetes insipidus, which occurred in all patients with replacement therapy. These patients might benefit from the administration of aqueous vasopressin by continuous infusion to control the fluid and electrolyte balance as previously reported (Bryant et al, 1994). Only one patient developed life-threatening infection leading to interruption of chemotherapy. By contrast, the first international CNS GCT study group, using an intensive carboplatin–etoposide and bleomycin combination, has reported significant regimen-associated morbidity and mortality (Balmaceda et al, 1996). The aim of that protocol was different, in trying to avoid radiotherapy for responding patients. There, half of the 45 germinoma patients achieved long-term remission without radiotherapy, but 22 required salvage treatment including extensive radiotherapy and/or high-dose chemotherapy for some patients. Seven patients died, four from toxicity and three from progression. No prognostic factor was identified that could distinguish the group of patients who required only chemotherapy. In our study, all patients were given radiotherapy, regardless of their response to chemotherapy. Since chemotherapy has been shown to be effective in intracranial germinoma, the decision to irradiate responding patients may be debatable. The difference in terms of tumour control between chemotherapy alone and chemotherapy followed by focal radiotherapy favours the second option. The low progression rate in our report is qualified by a short follow-up in a disease where late recurrences are not uncommon (Ono et al, 1994). However, the 42-month follow-up for the whole group does not differ from other large prospective series (Gobel et al, 1993; Balmaceda et al, 1996). Chemotherapy in our experience did allow avoidance of prophylactic craniospinal radiotherapy in patients with localized germinomas. Four patients relapsed, including two with spinal seeding. One initially non-secreting patient had elevated βHCG at recurrence, which compares to previous reports on recurrent intracranial germinomas (Ono et al, 1994; Balmaceda et al, 1996). The failure rate is low (7%) and three patients achieved complete remission following salvage treatment. Only one patient, who had metastatic disease at presentation, died from disease progression. The 3-year event-free survival (96%) and the overall survival (98%) compare favourably with results from other series.

The long-term side-effects of carboplatin, ifosfamide and etoposide are well described. The main potential issue of this protocol concerns fertility in males and the risk of secondary leukaemia. The cumulative doses of ifosfamide and etoposide are 18 g $m^{-2}$ and 1800 mg $m^{-2}$, respectively. The risk of ifosfamide-associated infertility in males is still poorly documented and more follow-up is required to know whether such a dose leads to gonadal late effects. The risk of secondary etoposide-induced leukaemia is dose-dependent and most of the cases reported previously occurred with cumulative doses above 2 g $m^{-2}$ (Smith et al, 1994). However, this risk has been considered in the design of future studies and the forthcoming SFOP protocol is planned with a reduced cumulative dose of 1.2 g $m^{-2}$ of etoposide. In this series, the follow-up is too short to assess the fertility in survivors, and no patient has developed secondary malignancy. The fertility question is complicated by the high incidence of tumour- or surgery-induced pituitary damage, especially among patients with suprasellar germinomas.

In this series, patients have been arbitrarily divided in two groups: those with genuine metastatic disease (six patients), and those with bifocal lesions (seven patients, one patient having a bifocal lesion with metastatic deposits). Low-dose craniospinal radiotherapy after chemotherapy led to prolonged remission in five out of six patients with genuine metastatic disease. Local radiotherapy to the primary site led to 100% control in patients with non-metastatic bifocal lesions. This suggests that bifocal lesions may be considered as loco-regional disease rather than metastatic, and that the need for craniospinal radiotherapy can be limited to those patients with spinal seeding only. This points out the importance of the initial staging, including CSF cytology and MRI scan of the spine, when treatment modalities vary according to the extent of the disease.

Management in intracranial germinoma benefits from the chemo- and radiosensitivity of this tumour. For decades, paediatric neuro-oncologists have been looking for alternatives to craniospinal radiotherapy in malignant brain tumours. While craniospinal radiotherapy is still a prerequisite for cure in medulloblastoma, in germinoma chemotherapy can be utilized to achieve dose and field reduction. One may question whether local radiotherapy only should be an alternative to combined chemo-radiotherapy. Information on this issue is limited and ultimately only randomized studies comparing chemotherapy combined with radiotherapy to local radiotherapy alone will answer this question. The dose of radiotherapy and the extent of the fields are other important issues. The 40 Gy dose might be reduced as well as the volume irradiated, considering the frequent massive shrinkage observed after chemotherapy in germinomas. Oncologists have two treatment modalities that achieve the same excellent survival rate. Long-term follow-up studies reporting on quality of life for survivors are required to draw definite conclusions on the optimal management with minimum side-effects.

### ACKNOWLEDGEMENTS

We thank the Paediatric Oncologists, Radiotherapists and Neurosurgeons from the 25 centres who collaborated to this SFOP study.

### REFERENCES

Allen JC, Bosl G and Walker R (1985) Chemotherapy trials in recurrent primary intracranial germ cell tumors. *J Neuro-oncol* **3**: 147–152

Allen JC, Kim JH and Packer JR (1987) Neoadjuvant chemotherapy for newly diagnosed germ-cell tumors of the central nervous system. *J Neurosurg* **67**: 65–70

Allen JC, Darosso R, Donahue B and Nirenberg A (1994) A phase II trial of preirradiation carboplatin in newly diagnosed germinoma of the central nervous system. *Cancer* **74**: 940–944

Balmaceda C, Heller G, Rosenblum M, Diez B, Villablanca JG, Kellie S, Maher P, Vlamis V, Walker RW, Leibel S and Finlay JL (1996) Chemotherapy without irradiation – a novel approach for newly diagnosed CNS germ cell tumors: results of an international cooperative trial. *J Clin Oncol* **14**: 2908–2915

Baranzelli MC, Patte C, Bouffet E, Couanet D, Habrand JL, Portas M, Lejars O, Lutz P, Le Gall E and Kalifa C (1997) Non-metastatic intracranial germinomas. The experience of the French Society of Pediatric Oncology. *Cancer* **80**: 1792–1797

Brada M and Rajan B (1990) Spinal seeding in cranial germinomas. *Br J Cancer* **61**: 339–340

Bryant WP, O'Marcaigh AS, Ledger GA and Zimmerman D (1994) Aqueous vasopressin infusion during chemotherapy in patients with diabetes insipidus. *Cancer* **74**: 2589–2592

Calaminus G, Bamberg M, Baranzelli MC, Benoit Y, di Montezemolo LC, Fossati Bellani F, Jurgens H, Kuhl HJ, Lenard HG and Curto ML (1994) Intracranial germ cell tumors: a comprehensive update of the European data. *Neuropediatrics* **25**: 26–32

Clayton PE and Shalet SM (1991) The evolution of spinal growth after irradiation. *Clin Oncol R Coll Radiol* **3**: 220–222

Darendeliler F, Livesey EA, Hindmarsh PC and Brook CG (1990) Growth and growth hormone secretion in children following treatment of brain tumours with radiotherapy. *Acta Paediatr Scand* **79**: 950–956

© Cancer Research Campaign 1999

Dattoli MJ and Newall J (1990) Radiation therapy for intracranial germinoma: the case for limited volume treatment. *Int J Radiation Oncol Biol Phys* **19**: 429–433
Dearnaley DP, A'Hern RP, Whittaker S and Bloom HJ (1990) Pineal and CNS germ cell tumors: Royal Marsden Hospital experience 1962–1987. *Int J Radiat Oncol Biol Phys* **18**: 773–781
Gentet JC, Doz F, Bouffet E, Plantaz D, Roche H, Tron P, Kalifa C, Mazingue F, Sariban E, Chastagner P, Bernard JL, Brunat-Mentigny M, Raybaud C and Zucker JM (1994) Carboplatin and VP 16 in medulloblastoma: a phase II study of the French Society of Pediatric Oncology (SFOP). *Med Ped Oncol* **23**: 422–427
Gobel U, Bamberg M, Budach V, Haas RJ, JankaSchaub G, Kuhl J, Lenard HG, Rister M and Spaar HJ (1989) Intracraniale keimzelltumoren: analyse der therapiestudie MAKEI 83/86 und protokollanderungen fur die nachfolgesstudie. *Klinische Pädiatrie* **201**: 261–268
Gobel U, Bamberg M, Calaminus G, Gnekow AK, Herrmann HD, Lenard HG, Spaar HJ, Niethammer D, Kuhl J and Harms D (1993) Verbesserte prognose intracranialer keimzelltumoren durch Intensivierte therapie: ergebnisse des therapieprotokolls MAKEI 89. *Klinische Pädiatrie* **205**: 217–224
Haddock MG, Schild SE, Scheithauer BW and Schomberg PJ (1997) Radiation therapy for histologically confirmed primary central nervous system germinoma. *Int J Radiat Oncol Biol Phys* **38**: 915–923
Hardenbergh PH, Golden J, Billet A, Scott RM, Shrieve DC, Silver B, Loeffler JS and Tarbell NJ (1997) Intracranial germinoma: the case for lower dose radiation therapy. *Int J Radiat Oncol Biol Phys* **39**: 419–426
Heideman RL, Kovnar EH, Kellie SJ, Douglass EC, Gajjar AJ, Walter AW, Langston JA, Jenkins JJ, Li Y and Greenwald C (1995) Preirradiation chemotherapy with carboplatin and etoposide in newly diagnosed embryonal pediatric CNS tumors. *J Clin Oncol* **13**: 2247–2254
Kiltie AE and Gattamaneni HR (1995) Survival and quality of life of paediatric intracranial germ cell tumour patients treated at the Christie Hospital, 1972–1993. *Med Pediatr Oncol* **25**: 450–456
Kobayashi T, Yoshida J, Ishiyama J, Noda S, Kito A and Kida Y (1989) Combination chemotherapy with cisplatin and etoposide for malignant intracranial germ cell tumors. An experimental and clinical study. *J Neurosurg* **70**: 676–681
Linstadt D, Wara WM, Edwards MSB, Hudgins RJ and Sheline GE (1988) Radiotherapy of primary intracranial germinomas: the case against routine craniospinal. *Int J Radiat Oncol Biol Phys* **15**: 291–297
Manera L, Regis J, Chinot O, Porcheron D, Levrier O, Farnarier P and Peragut JC (1996) Pineal region tumors: the role of stereotactic radiosurgery. *Stereotact Funct Neurosurg* **66** (Suppl 1): 164–173
Matsumani M, Sano K, Takakura K, Fujimaki T, Nakamura O, Funata N and Seto T (1997) Primary intracranial germ cell tumors: a clinical analysis of 153 verified cases. *J Neurosurg* **86**: 446–455
Mostofi FK, Sester H and Davis CJ (1988) Development in histopathology of testicular germ cell tumors. *Semin Urol* **6**: 171–175
Mulhern RK, Hancock J, Fairclough D and Kun L (1992) Neuropsychological status of children treated for brain tumors: a critical review and integrative analysis. *Med Ped Oncol* **20**: 181–191
Munperow E and Hartmann M (1992) Spermatocitic cord beta human chorionic gonadotrophin levels in seminoma and their clinical implications. *J Urol* **147**: 1041–1043
Ninane J, Baurain R, de Kraker J, Ferster A, Trouet A and Cornu G (1989) Alkylating activity in serum, urine, and CSF following high-dose ifosfamide in children. *Cancer Chemother Pharmacol* **24** (Suppl 1): 2–6
Oi S and Matsumoto S (1992) Controversies pertaining to therapeutic modalities for tumors of the pineal region: a worldwide survey of different patient populations. *Childs Nerv Syst* **8**: 332–336
Ono N, Isobe I, Uki J, Kurihara H, Shimizu T and Kohno K (1994) Recurrence of primary intracranial germinomas after complete response with radiotherapy: recurrence patterns and therapy. *Neurosurgery* **35**: 615–621
Patel SR, Buckner JC, Smithson WA, Scheithauer BW and Groover RV (1992) Cisplatin-based chemotherapy in primary central nervous system germ cell tumors. *J Neuro-oncol* **12**: 47–52
Plowman PN, Kingston JE, Sebag-Montefiore D and Doughty D (1997) Clinical efficacy of perceived 'CNS friendly' chemoradiotherapy for primary intracranial germ cell tumours. *Clin Oncol R Coll Radiol* **9**: 48–53
Roth BJ (1996) The role of ifosfamide in the treatment of testicular and urothelial malignancies. *Semin Oncol* **23** (Suppl 7): 19–27
Sawamura Y, De Triboulet N, Ishii N and Abe H (1997) Management of primary intracranial germinomas: diagnostic surgery or radical resection? *J Neurosurg* **87**: 262–266
Sawamura Y, Shirato H, Ikeda J, Tada M, Ishii N, Kato T, Abe H and Fujieda K (1998) Induction chemotherapy followed by reduced-volume radiation therapy for newly diagnosed central nervous system germinoma. *J Neurosurg* **88**: 66–72
Shibamoto Y, Abe M, Yamashita J, Takahashi M, Hiroaoka M, Ono K and Tsutsui K (1988) Treatment results of intracranial germinoma as a function of the irradiated volume. *Int J Radiat Oncol Biol Phys* **15**: 285–290
Shibamato Y, Takahashi M and Abe M (1994) Reduction of the radiation dose for intracranial germinoma: a prospective study. *Br J Cancer* **70**: 984–989
Shibamoto Y, Takahashi M and Sasai K (1997) Prognosis of intracranial germinoma with syncytiotrophoblastic giant cells treated by radiation therapy. *Int J Radiat Oncol Biol Phys* **37**: 505–510
Shinoda J, Yamada H, Sakai N, Ando T, Hirata T and Miwa Y (1988) Placental alkaline phosphatase as a tumor marker for primary intracranial germinoma. *J Neurosurg* **68**: 710–720
Shirato H, Nishio M, Sawamura Y, Myohjin M, Katahara T, Nishioka T, Mizutani Y, Abe H and Miyasaka K (1997) Analysis of long-term treatment of intracranial germinoma. *Int J Radiat Oncol Biol Phys* **37**: 511–517
Smith MA, Rubinstein L and Ungerleider RS (1994) Therapy-related acute myeloid-leukemia following treatment with epipodophyllotoxins: estimating the risks. *Med Ped Oncol* **23**: 86–98
Sung OI, Harisiadis L and Chang CH (1978) Midline pineal tumors and suprasellar germinomas highly curable by irradiation. *Radiology* **128**: 745–751
Uematsu Y, Tsuura Y, Miyamoto K, Itakura T, Hayashi S and Komai N (1992) The recurrence of primary intracranial germinomas. Special reference to germinoma with STGC (syncytiotrophoblastic giant cell). *J Neurooncol* **13**: 247–256
Wolden SL, Wara WM, Larson DA, Prados MD, Edwards MS and Sneed PK (1995) Radiation therapy for primary intracranial germ-cell tumors. *Int J Radiat Oncol Biol Phys* **32**: 943–949

 *© Cancer Research Campaign 1999*
